# Medical insurance, vulnerability to poverty, and wealth inequality

**DOI:** 10.3389/fpubh.2024.1286549

**Published:** 2024-02-27

**Authors:** Xianhua Zhou, Xujin Yang

**Affiliations:** ^1^China Institute for Actuarial Science, School of Insurance, Central University of Finance and Economics, Beijing, China; ^2^School of Insurance, Central University of Finance and Economics, Beijing, China

**Keywords:** heterogeneous health risks, multi-equilibrium model, reverse redistribution, medical insurance, vulnerability to poverty, wealth inequality

## Abstract

**Background:**

China has made remarkable achievements in alleviating poverty under its current poverty standards. Despite these immense successes, the challenge of consolidating these achievements remains. In reality, health risks are among the significant factors causing rural households to fall into poverty, and medical insurance is the significant factor mitigating household vulnerability to poverty. Therefore, alleviating or guarding against households falling into poverty is essential.

**Methods:**

This paper establishes a multi-equilibrium model that incorporates heterogeneous health risks and medical insurance. Through parameter calibration and value function iteration, numerical solutions are derived.

**Results:**

Heterogeneous health risks significantly increase poverty vulnerability and wealth inequality in rural households. Medical insurance, through its investment incentives and loss compensation effects, efficiently mitigates these issues, especially benefiting those in poorer health. Furthermore, the dual-slanted compensation policy efficiently mitigates the adverse effects of “reverse redistribution.”

**Conclusion:**

Medical insurance effectively mitigates household vulnerability to poverty and wealth inequality. Government departments must establish health records for residents. By recognizing variations in health conditions, these departments can provide households with poorer health conditions with a higher medical expense compensation ratio. In addition, the government should further focus medical expense reimbursements toward households on the cusp of escaping poverty to ensure that they are not plunged back (or further) into poverty due to medical expenses.

## 1 Introduction

Poverty is one of the most pressing issues worldwide and remains a primary concern for development economists and policymakers. China has made remarkable achievements in alleviating poverty under its current poverty standards.^1^ By 2020, all 832 recognized poverty-stricken counties nationwide had been lifted out of poverty, cleared and nearly 100 million rural residents overcoming poverty. Remarkably, China achieved the United Nations’ 2030 Agenda for Sustainable Development’s poverty reduction goal a decade early, historically eradicating absolute poverty and creating an unprecedented feat in human poverty reduction history (Table 1).^2^

**Table 1:** Poverty-stricken counties, incidence of poverty and number of impoverished populations.

Year	Poverty-stricken counties	Incidence of poverty (%)	Impoverished populations (ten thousands)
2012	832	10.2	9,899
2013	832	8.5	8,249
2014	832	7.2	7,017
2015	832	5.7	5,575
2016	804	4.5	4,335
2017	679	3.1	3,046
2018	396	1.7	1,660
2019	52	0.6	551
2020	0	0	0

The positive correlation between health risks and household poverty has been discussed in several studies (1–3). Using household survey data from China, Song et al. (3) and Ma et al. (4) found that health risks are a direct factor leading to poverty vulnerability in households. Liao et al. (1) discovered that under the impact of health risks, increased household medical expenses lead to reductions in total household capital, total labor, and *per capita* capital, ultimately plunging households into poverty. Moreover, health risk shocks also generate health inequality and health poverty issues (2, 5) and can lead to the intergenerational transmission of household poverty (6, 7). This further exacerbates wealth inequality among households and ultimately heightens relative poverty within households (8). However, these studies rarely delve into the impact mechanisms of heterogeneous health risks on vulnerability to poverty and wealth inequality.

Several studies have deeply explored the impact of medical insurance on poverty alleviation. Participation in medical insurance can enhance a household’s nonmedical consumption (9), reduce excessive labor supply and out-of-pocket medical expenses (10, 11), improve residents’ health conditions (1, 12) and reduce mortality rates (13). Further studies show that participation in medical insurance can stabilize household income (10) and enhance social welfare (14). More importantly, such participation can reduce the likelihood of households falling into poverty due to health risk shocks (1, 15–17) and the income gap between urban and rural residents (18). Korenman et al. (19) developed a health-inclusive poverty measure and found that participation in medical insurance reduced the poverty rate by 2.9 percentage points among people under 65 in Massachusetts and by 3.2 percentage points among children. However, if the coverage of medical insurance is too low, it is less effective (20).

Van Doorslaer et al. (21) found that approximately half of OECD countries experience unbalanced utilization of medical services, with higher-income groups benefiting more. Similar conclusions were also drawn from studies using medical insurance data from Europe, the United States, and Asian countries by van Doorslaer et al. (22) and Lu et al. (23). These studies indicate that despite medical insurance effectively reducing poverty vulnerability and income disparities, it also causes the “reverse redistribution” of wealth. Using health insurance data from Massachusetts, Finkelstein et al. (24) found that a slanted compensation policy could increase participation rates among low-income groups, providing some fuel for addressing the “reverse redistribution” issue.

Therefore, building on these studies, this paper incorporates heterogeneous health risks and medical insurance into a multiple equilibrium model^3^ and discusses the impact of heterogeneous health risk shocks, medical insurance, and dual-slanted compensation policies on rural household poverty vulnerability and wealth inequality.

The potential marginal contributions of this paper are as follows: First, this paper introduces heterogeneous health risks into the multiple equilibrium model, discussing the impact of heterogeneous health risks on rural household poverty vulnerability and wealth inequality. Second, based on the investment incentive effect of medical insurance and the “reverse redistribution” effect of wealth, this paper discusses the impact of basic medical insurance on household vulnerability to poverty and wealth inequality. Third, to address the “reverse redistribution” issue in medical insurance, we designed a dual-slanted compensation policy based on wealth and health status to further optimize the effect of medical insurance on reducing poverty vulnerability and wealth inequality.

The rest of this research is structured as follows. The next section presents the methodology. The third section provides the results and discussion. The conclusions and policy recommendations are offered in the last section.

## 2 Methods

### 2.1 Multi-equilibrium model

#### 2.1.1 Production function

Assume that each household possesses two types of agricultural production technologies, high and low. If the productive asset $k_{t}\leq \bar{k}$ , the farmer opts for the technology with lower production efficiency. If $k_{t}>\bar{k}$ , the farmer chooses the technology with higher production efficiency. $\bar{k}$ denotes the asset threshold for the technology switch. Hence, the non-convex agricultural production function is as follows:


(1) $$f\left(\;k_{t}\right)=\{\begin{array}{c} f_{H}\left(k_{t}\right)=A_{t}k_{t}^{\alpha_{H}}-{\bar{f}}_{H},k_{t}>\bar{k}\\ f_{L}\left(k_{t}\right)=A_{t}k_{t}^{\alpha_{L}}-{\bar{f}}_{L},k_{t}\leq \bar{k} \end{array}$$

where $A_{t}$ denotes the total factor productivity in agriculture. ${\bar{f}}_{H}$ and ${\bar{f}}_{L}$ are the fixed costs associated with the high and low production technologies, respectively. They are also the reasons behind poverty traps (30, 32).^4^ $\alpha_{H}$ and $\alpha_{L}$ represent the capital output elasticity for high and low production technologies, respectively. The capital technology switch threshold is the intersection of the outputs for both agricultural production technologies, that is:


$$\bar{k}=\left\{k|f_{H}\left(k_{t}\right)=f_{L}\left(k_{t}\right)\right\}$$

#### 2.1.2 Intertemporal household decision model

We assume an infinite number of homogeneous small-scale farming households, with a fixed family size standardized to 1. Household are immortal and aim to maximize their utility. Each household produces only one homogenous agricultural product. The capital for agricultural production comes internally from the household. The household decides on consumption before making agricultural production decisions, and the initial capital for the household is $k_{0}$ . The discount factor for household utility is β , and the depreciation rate of the household’s assets is δ . Therefore, the objective of maximizing household utility is:


(2) $$\underset{c_{t}}{\max}E_{\theta }\;\sum_{t=0}^{\infty }\beta^{t}u\left(c_{t}\right)$$

where $u\left(c_{t}\right)=\frac{c_{t}^{1-\gamma }}{1-\gamma }\;$ represents the constant relative risk aversion (CRRA) power utility function, $c_{t}$ denotes the household’s consumption in period t , and γ is the coefficient of risk aversion. This is subject to the following constraints:


$$c_{t}\leq k_{t}+f\left(k_{t}\right)$$

$$k_{t+1}=\left(f\left(k_{t}\right)+\left(1-\delta \right)k_{t}-c_{t}-\left(1-s_{t}\right)m_{t}\right)\left(1-\theta_{i,t+1}+I_{t+1}B_{t+1}\right)$$

$$\;0\leq m_{t}\leq A_{t}$$

$$c_{t},k_{t}>0.$$

The first constraint represents the liquidity constraint on wealth (34), implying that households cannot borrow for consumption or investment. The second constraint is the motion equation for productive assets. $\theta_{i,t+1}$ represents the ratio of total medical expenses paid by the household to its assets, i∈[b,g] . b and g denote individuals with poor and good health, respectively. Those with good health have to pay less in medical expenses when confronted with health risk, and $0\leq \theta_{g,t}\leq \theta_{b,t}\leq 1$ . The probability of health risk is $p_{i,t}$ , and $0\leq p_{g,t}\leq p_{b,t}\leq 1$ , with health risk being independently and identically distributed. $I_{t}$ indicates the indicator function for the occurrence of a health risk, $B_{t}=\left(1-\theta_{i,t}\right)\eta_{t}$ represents the ratio of compensation received to assets, and $\eta_{t}$ denotes the reimbursement rate, which can also be interpreted as the level of medical insurance coverage. $s_{t}$ stands for the government subsidy rate for medical insurance premiums, and $\left(1-s_{t}\right)m_{t}$ represents the medical insurance fees that rural households have to pay themselves. The third constraint suggests that the price of the insurance premium should be greater than or equal to 0 but cannot exceed the total assets of the household. The fourth constraint indicates that both consumption and assets must be positive values.

Based on Eq. 2, and given the state variable $k_{t}$ , the Bellman equation for utility maximization can be derived:


(3) $$V\left(k_{t}\right)=\underset{c_{t}}{\max}\left\{u\left(c_{t}\right)+\beta E_{\theta_{i,t}}\left[V\left(k_{t+1}\right)|c_{t},k_{t}\right]\right\}$$

where $V\left(k_{t}\right)$ represents the value function.

Based on Eqs 2, 3, we derive the first-order condition for the household, illustrating the intertemporal trade-off between consumption and investment as follows:


(4) $$u^{\prime }\left(c_{t}\right)\geq \beta E_{\theta }\left[\;V^{\prime }\left(k_{t+1}\right)\left(1-\theta_{i,t+1}\right)\right]=\lambda \left(k_{t+1}\right)$$

where $V^{\prime }\left(k_{t+1}\right)$ represents the future value of a unit of capital, and $\lambda \left(k_{t+1}\right)$ denotes the expected shadow price of the asset.

#### 2.1.3 Medical insurance

The current Urban and Rural Residents’ Basic Medical Insurance in China follows a model combining individual payments with government subsidies.^5^ Individuals contribute the same amount toward medical insurance premiums, and when confronted with health risk, they receive compensation for medical expenses at the same rate. It is posited that the medical insurance premium is priced based on the principle of expected value, which is:


(5) $$m_{t}=\eta_{t}\left(1-d\right)\left(\varepsilon_{b,i}\theta_{b,t}p_{b,t}+\varepsilon_{g,i}\theta_{g,t}p_{g,t}\right)\frac{1}{N}\sum_{n=1}^{N}W_{n,t}$$

where $W_{n,t}=f\left(k_{n,t}\right)+\left(1-\delta \right)k_{n,t}-c_{n,t}-\left(1-s_{t}\right)m_{t}$ represents the family’s wealth after making consumption decisions, $\varepsilon_{i,i}$ denotes the proportion of individuals in healthy and unhealthy states, and N stands for the number of insured individuals.

### 2.2 Parameter calibration

For total factor productivity $A_{t}$ , we set $A_{t}=1$ . According to Liao et al. (26), the capital output elasticity for high and low agricultural production techniques are set at $\alpha_{H}=0.5$ and $\alpha_{L}=0.1$ , respectively. The utility discount rate is set at β=0.975 . The capital depreciation rate δ is 0.096. According to Liao et al. (26), the risk aversion coefficient is set to γ=0.53 . For individuals in good health and those in poorer health, the asset loss ratios are set at $\theta_{g,t}=0.2$ and $\theta_{g,t}=0.4$ , respectively. The proportion of the population in good health compared to that in poorer health is $\varepsilon_{g,t}=0.72$ and $\varepsilon_{b,t}=0.28$ , respectively. Furthermore, the probability of encountering a health risk for individuals in good health versus those in poor health are set at $p_{g,t}=0.025$ and $p_{b,t}=0.05$ , respectively. We set the deductible rate as d=0 .

According to the actual reimbursement rate data for urban and rural resident medical insurance, released by China’s National Medical Security Administration over the past 3 years,^6^ this paper estimates an average actual reimbursement rate $\eta_{t}=0.6$ . The government subsidy rate for the medical insurance of urban and rural residents is approximately $s_{t}=0.7$ , as referenced by Liu (14). In this study, the fixed costs for high and standard production technologies are set at ${\bar{f}}_{H}=0.95$ and ${\bar{f}}_{L}=0$ , respectively.

### 2.3 Value function iteration method

Building upon the aforementioned parameter calibration, this study further employs the value function iteration method to compute the policy functions for consumption and capital. That is, we calculate the present consumption and the subsequent capital value for each initial capital level. Given that in the theoretical model, the health risk occurs after the household’s current consumption decision but before the next period’s consumption decision, we designate the consumption $c_{t}$ of the t period as the control variable. The detailed computational steps are as follows:

First, in period t , based on state variable $k_{t}$ , we determine the initial range of control variable $c_{t}\in \left[0,f\left(k_{t}\right)+k_{t}-\left(1-s_{t}\right)m_{t}\right]$ . For simplicity, we set the initial value series of the value function $V\left(k_{t}\right)$ to 0. We then define the range for the state variable $k_{t}$ in period t , ensuring $k_{t}\in \left[k_{\min},k_{\max}\right]$ . We set $k_{\min}=0.1$ and $k_{\max}=20$ .

Second, by utilizing the motion equation of capital and the loss distribution from health risk shocks, we identify the initial capital level for the next period. By applying linear interpolation techniques, we establish a one-to-one mapping relationship between the capital in period t+1 and the values of the value function sequence $V\left(k_{t+1}\right)$ . Together with the loss distribution from health risk, we obtain the value of the value function $V\left(k_{t}\right)$ corresponding to the state variable $k_{t}$ .

Third, the state variable $k_{t}$ is divided into 100 intervals. We obtain the consumption sequence $c_{t}$ that maximizes utility, as well as the sequence for the value function. The iteration halts when the percentage change between two consecutive value functions $V\left(k_{t}\right)$ is less than $10^{-10}$ .

Fourth, based on the distribution of losses due to health risk and the motion equation for capital, we determine the policy function value for capital $k_{t+1}$ .

Finally, by making 100,000 random simulations and modeling 100,000 rural households, we compute a range of results, including the Micawber threshold, vulnerability to poverty among rural households, and wealth inequality.

Notably, this study utilizes the fminbnd function from the Optimization Toolbox in MATLAB to compute the utility-maximizing value function $V\left(k_{t}\right)$ and its corresponding policy function. Furthermore, regarding the calculation of medical insurance premiums, we rely on Eq. 5. Considering the distribution of household wealth status in Table 2, we simulate 100,000 households, from which we ultimately calculate the average medical insurance premium.

**Table 2:** Proportion of households at different asset levels.

Asset	0–1	1–2	2–3	3–4	4–5
Proportion (%)	5.6	7.5	9	9.8	9.3
Asset	5–6	6–7	7–8	8–9	9–10
Proportion (%)	8.7	7.6	6.6	5.6	4.8
Asset	10–11	11–12	12–13	13–14	14–15
Proportion (%)	3.9	3.2	2.8	2.4	1.9
Asset	15–16	16–17	17–18	18–19	19–20
Proportion (%)	1.7	1.4	1.2	1	6

## 3 Results and discussion

### 3.1 Vulnerability to poverty and wealth inequality under heterogeneous health risks

Based on the model construction and value function iteration from the second section, we discuss the changes in rural household vulnerability to poverty and wealth inequality under heterogeneous health risk in this section. We measure poverty vulnerability by the probability that a rural household’s assets fall below the Micawber threshold.

**Figure 1:**
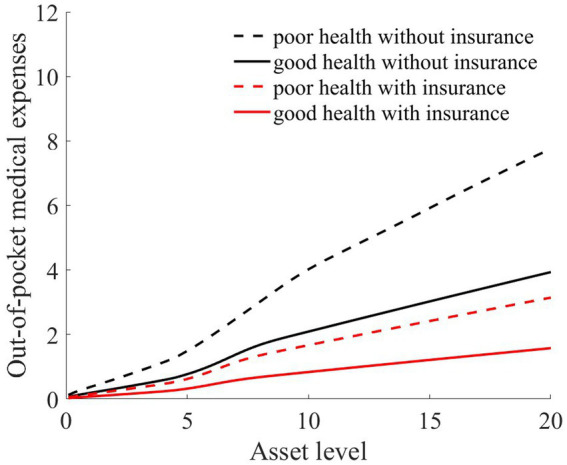
Out-of-pocket medical expenses after health risk.

**Figure 2:**
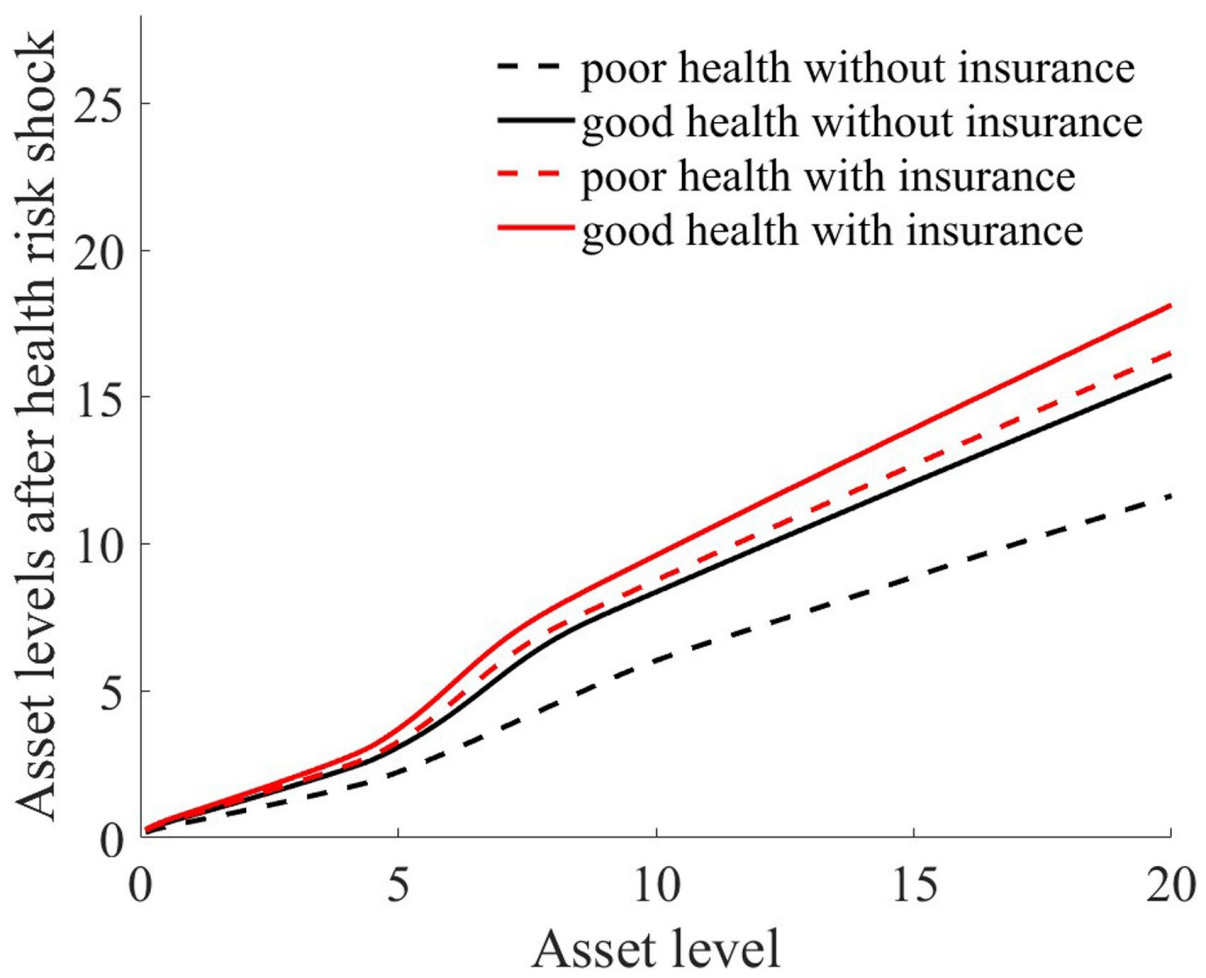
Asset levels after health risk.

**Figure 3:**
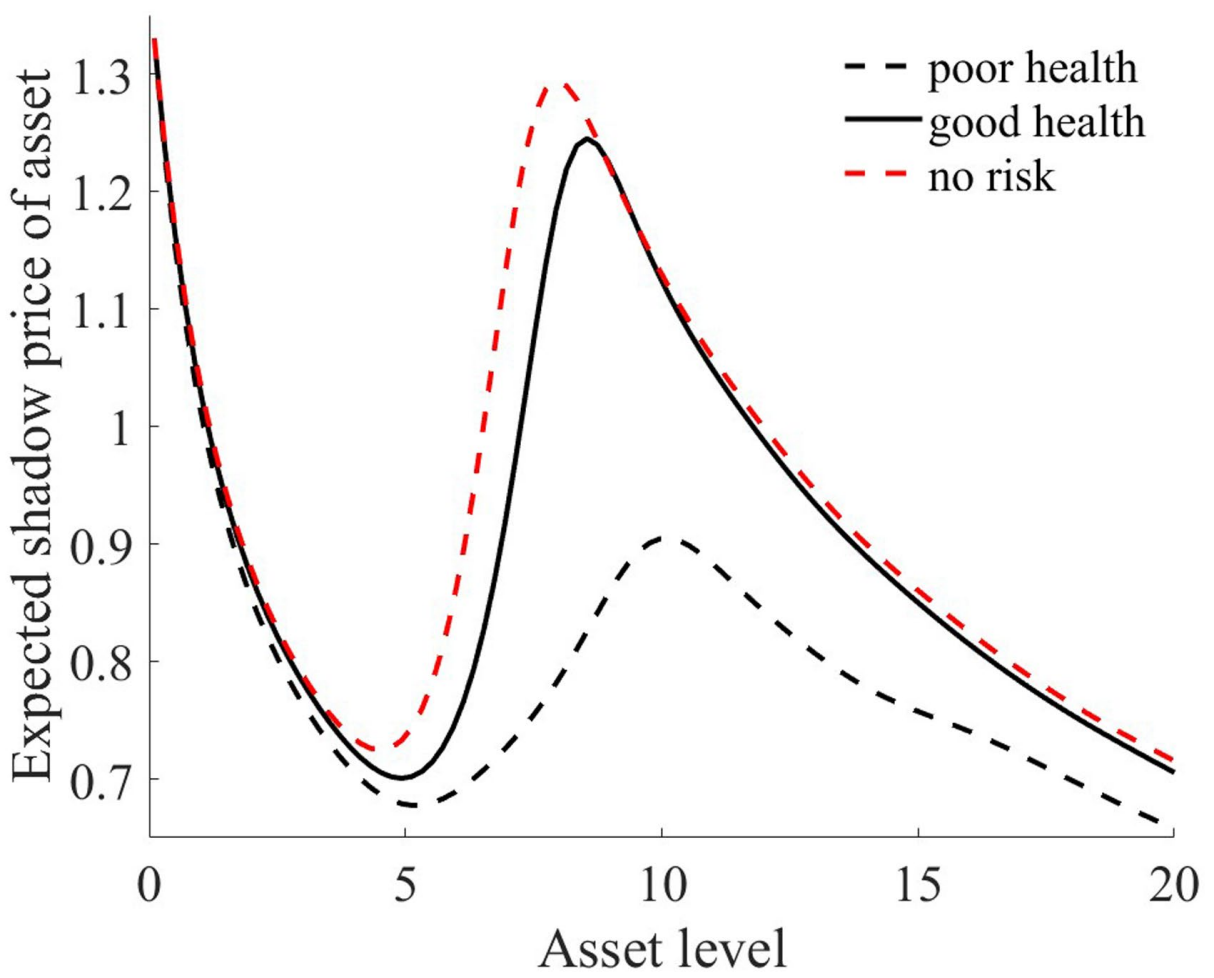
Shadow prices of assets.

**Figure 4:**
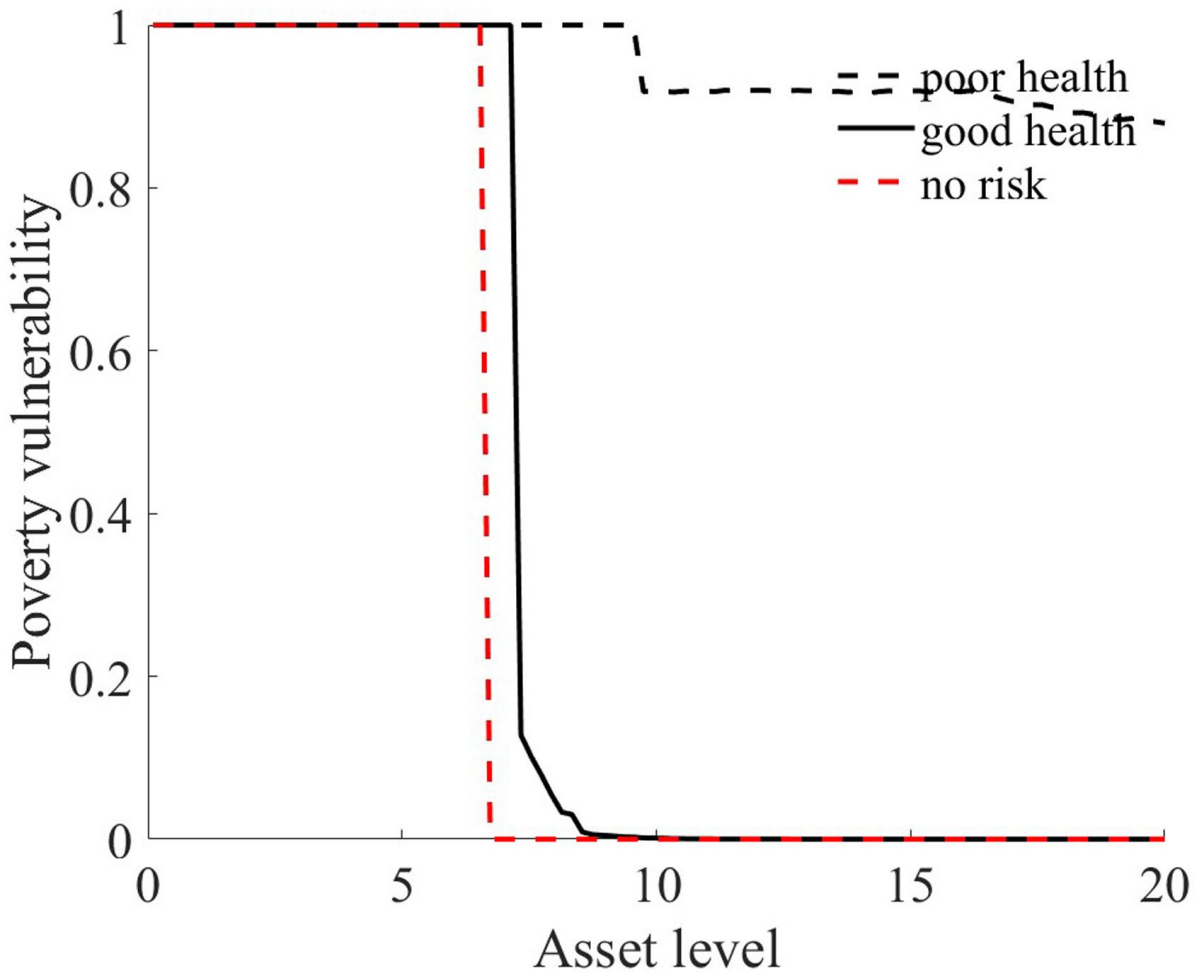
Poverty vulnerability.

As illustrated in Figures 1–4, we simulated household out-of-pocket medical expenses and asset levels using Eqs 1–3. We then calculated the shadow prices of assets through Eq. 4 and simulated the vulnerability to poverty for rural households over the next 50 periods. Additionally, we presented scenarios without health risk. The solid black line represents households with good health, the dashed black line indicates households with poor health, and the dashed red line denotes households without health risk. For any given household, the higher the asset level is, the lower the vulnerability to poverty. This is not only because households with higher asset levels possess greater resilience against risks but also because these households’ assets are farther from the Micawber threshold. Moreover, after encountering health risks, there is a decline in the shadow prices of assets (as shown in Figure 3), which reduces household investments in agricultural production, thereby increasing these households’ vulnerability to poverty.

Households with better health face fewer health risks in the future than households with poor health. As a result, they incur lower medical expenses (as depicted in Figure 1) and, upon each health risk, experience a smaller proportion of asset loss (Figure 2). Furthermore, even though the shadow price of assets declines after health risk, it remains higher than that of households with poor health (Figure 3). This suggests that they continue to maintain a higher level of investment in agricultural production, anticipating higher future consumption. Due to the stronger investment incentive effect, they are more likely to reach a high steady-state equilibrium. This leads to a considerably lower Micawber threshold (7.0109) for these households than for households with poor health (9.1459). Consequently, households with good health have less vulnerability to poverty than households with poorer health (Figure 4).

In contrast, for households with poorer health, each time they suffer from a health risk, their remaining assets tend to be lower, positioning them closer to the Micawber threshold. Moreover, the shadow price of their assets drops significantly (as illustrated in Figure 3), which inhibits their investment in agricultural production. These households also face a higher Micawber threshold. As a result, these households are more inclined to allocate their wealth toward immediate consumption rather than future consumption, increasing their probability to falling into the poverty trap (as depicted in Figure 4).

Having previously discussed the differentiated vulnerability to poverty under heterogeneous health risks, it is also evident that households with high asset levels and those with low asset levels display distinct vulnerabilities. We further delve into the shifts in wealth inequality. Assuming that wealth inequality initially exists among households, after encountering health risks, households with poorer health invariably face higher medical expenses (as demonstrated in Figure 1). This could lead to an even greater divergence in the asset levels of different households, potentially exacerbating the wealth disparity between them.

Adopting the approach of Cagetti and De Nardi (35), we employ the Gini coefficient to measure wealth inequality. Additionally, to further test the robustness of these results, we use the Theil index as a measure of wealth disparity.

Liao et al. (26), according to the rural household income distribution data published by the National Bureau of Statistics of China for 2012, segmented households into 20 groups from lowest to highest income, detailing the proportion of rural households in each segment. Following this approach, we set the household asset range from 0 to 20, with asset intervals equally distributed. The overall proportions of households with different assets are presented in Table 2.

**Figure 5:**
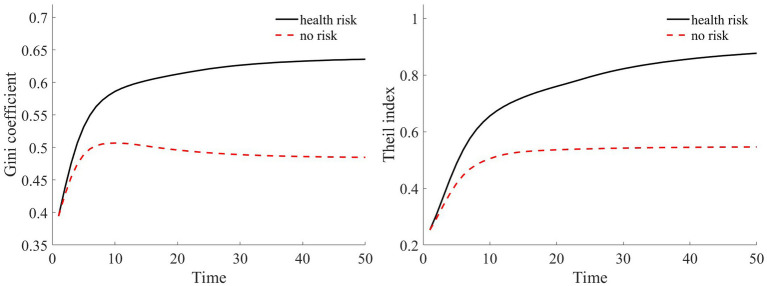
Changes in wealth inequality indices.

In our assumption, rural households at varying wealth levels comprise both households in good health and households in poor health. Furthermore, both households with good health and households with poor health are evenly distributed across the different asset groups. We simulate a total of 100,000 households over a span of 50 years. Based on Table 2, we calculate the composition of households in each asset range. For every simulated household, their health status was determined via random sampling. Through this random simulation, we present the Gini coefficient and Theil index under health risk shocks in Figure 5.

Compared to scenarios without risk, when exposed to health risk, households experience a decline in the shadow price of assets (as illustrated in Figure 3). Households tend to use their assets more for consumption than for production investment. This leads to a greater number of households falling into poverty traps, exacerbating the degree of wealth disparity. Households with good health conditions face lower health risks. Although the shadow price of their assets diminishes, it remains considerably higher than that of households with poor health conditions (as shown in Figure 3). They continue to maintain substantial agricultural asset investments that allow rapid capital accumulation, thereby approaching or achieving a high steady-state equilibrium.

Conversely, for households with poor health conditions, consistent exposure to health risk results in a lower expected shadow price for assets. These households are more inclined toward immediate consumption, making them more susceptible to falling into poverty traps. Over time, this disparity in wealth intensifies, leading to a continual expansion in both the Gini coefficient and Theil index (as shown in Figure 5). This indicates that the degree of wealth inequality progressively increases as health risk increases.

### 3.2 Impact of medical insurance on the vulnerability to poverty among rural households

By employing value function iteration, we derived the policy function for household assets and computed the out-of-pocket medical expenses for households, as well as their assets.

As illustrated in Figures 1, 2, the solid black line represents households with good health conditions that are not enrolled in medical insurance, while the dashed black line signifies households with poor health conditions without medical insurance. Conversely, the solid red line denotes households with good health conditions that have medical insurance, and the dashed red line corresponds to households with poorer health conditions that have medical insurance. Broadly speaking, regardless of insurance enrollment, households with worse health conditions incur higher medical expenses. Moreover, households with more robust economic standing are better equipped to shoulder these increased medical expenses.

As evidenced by Figure 1, medical insurance, through substantial medical expense reimbursements, has alleviated the healthcare burden on rural households. Given that the reimbursement rate is consistent, higher medical expenditures lead to greater reimbursements. This is particularly significant for individuals with poorer health, where medical insurance has considerably reduced the medical financial strain on rural households.

As illustrated in Figure 2, regardless of whether households have good or poor health conditions, after basic medical insurance compensation is received, the out-of-pocket medical expenses significantly decline. Consequently, the proportion of asset losses for rural households that receive medical compensation is substantially reduced. This, in turn, elevates the asset levels for the subsequent period, playing a crucial role in wealth accumulation for rural households. Comparatively, medical insurance provides higher compensation for households in poorer health.

The aforementioned results highlight that medical insurance has played an effective role in risk compensation, offering significant support for stabilizing consumption and fostering wealth accumulation for rural households. Furthermore, we compared the changes in vulnerability to poverty across four types of rural households: those in good health without medical insurance, those in poor health without medical insurance, those in good health with medical insurance, and those in poor health with medical insurance.

**Figure 6:**
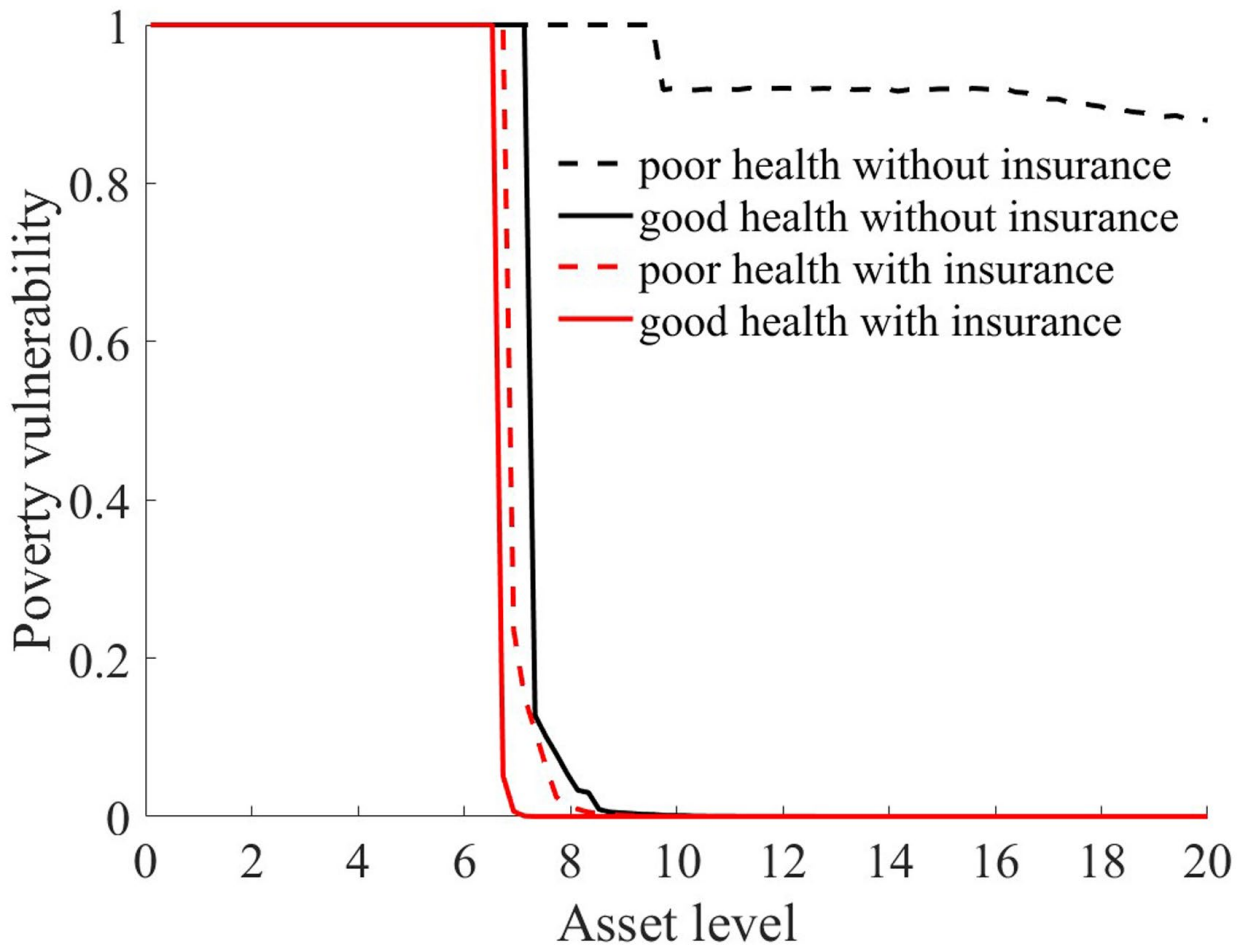
Poverty vulnerability.

**Figure 7:**
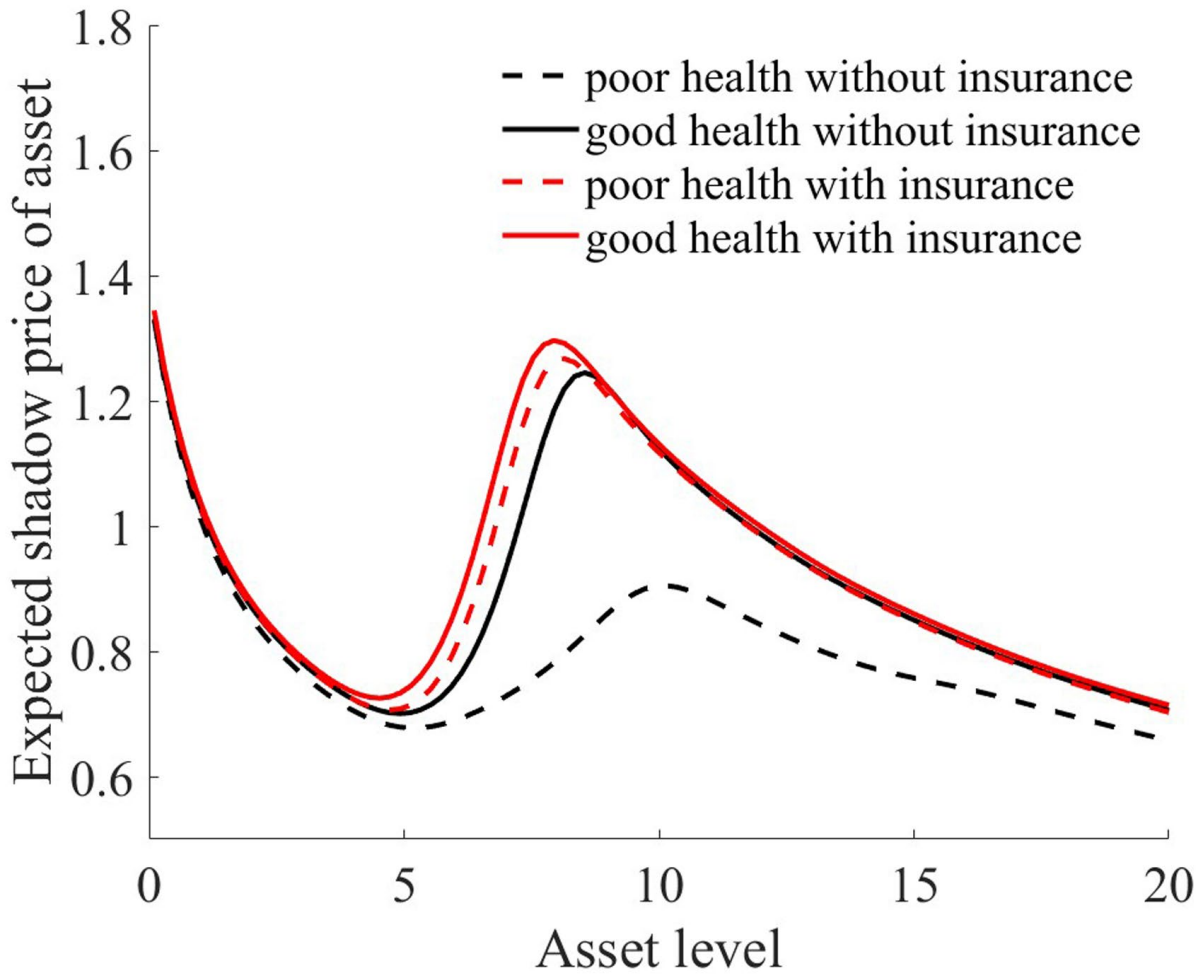
Shadow prices of assets.

As illustrated in Figure 6, rural households, regardless of whether they have good or poor health conditions, have significantly reduced their vulnerability to poverty upon enrollment in medical insurance. This finding underscores the role of medical insurance in mitigating the risk of rural households falling into poverty due to health risk. The reason is that rural households with medical insurance, when facing health risk, can reduce their out-of-pocket medical expenses through insurance compensation, thereby enhancing their post-shock asset levels (as shown in Figure 2). Moreover, due to the risk-protection feature of medical insurance, the shadow price of household assets increases (as shown in Figure 7). Compared to those without medical insurance, this encourages households to engage in more productive investments, aiming for greater future consumption. This notably diminishes the vulnerability of rural households to poverty.

From the perspective of households with varying health conditions, medical insurance appears to be more beneficial for those in poorer health. This is reflected not only in the reduction in out-of-pocket medical costs for these households following health risk (as shown in Figure 1) but also in the increase in the shadow price of assets for households with poorer health conditions (as indicated in Figure 7). This markedly reduces the vulnerability to poverty for households with poorer health conditions (Figure 6). Of course, since all households are required to pay a certain medical insurance premium, these premiums have an erosive effect on assets, which can mitigate the overall impact of the insurance. Overall, medical insurance significantly diminishes the vulnerability to poverty among rural households.

### 3.3 The impact of medical insurance on wealth inequality

In our previous discussions, we delved into the impact of medical insurance on vulnerability to poverty. However, under an egalitarian compensation system, it becomes imperative to further discuss the fairness of individual benefits from medical insurance.

Public medical service benefits are often measured through the Benefit Incidence Analysis framework, and individuals with different wealth statuses tend to use public medical services differently (36). Therefore, referencing the measurement method of O’Donnell et al. (36), the expression for individual benefits from public medical services is as follows:


(6) $$b_{t}^{n}=\frac{e_{t}}{\sum_{n=1}^{N}u_{t}^{n}}u_{t}^{n},e_{t}=Nm_{t}s_{t}$$

(7) $$u_{t}^{n}=\left(f\left(k_{t}\right)+\left(1-\delta \right)k_{t}-c_{t}-\left(1-s_{t}\right)m_{t}\right)I_{t+1}B_{t+1}$$

where $e_{t}$ represents the government’s investment in public medical services, and $u_{t}^{n}$ denotes the quantity of public medical services used by the n -th household in period t (that is, the medical reimbursement expenses).

**Figure 8:**
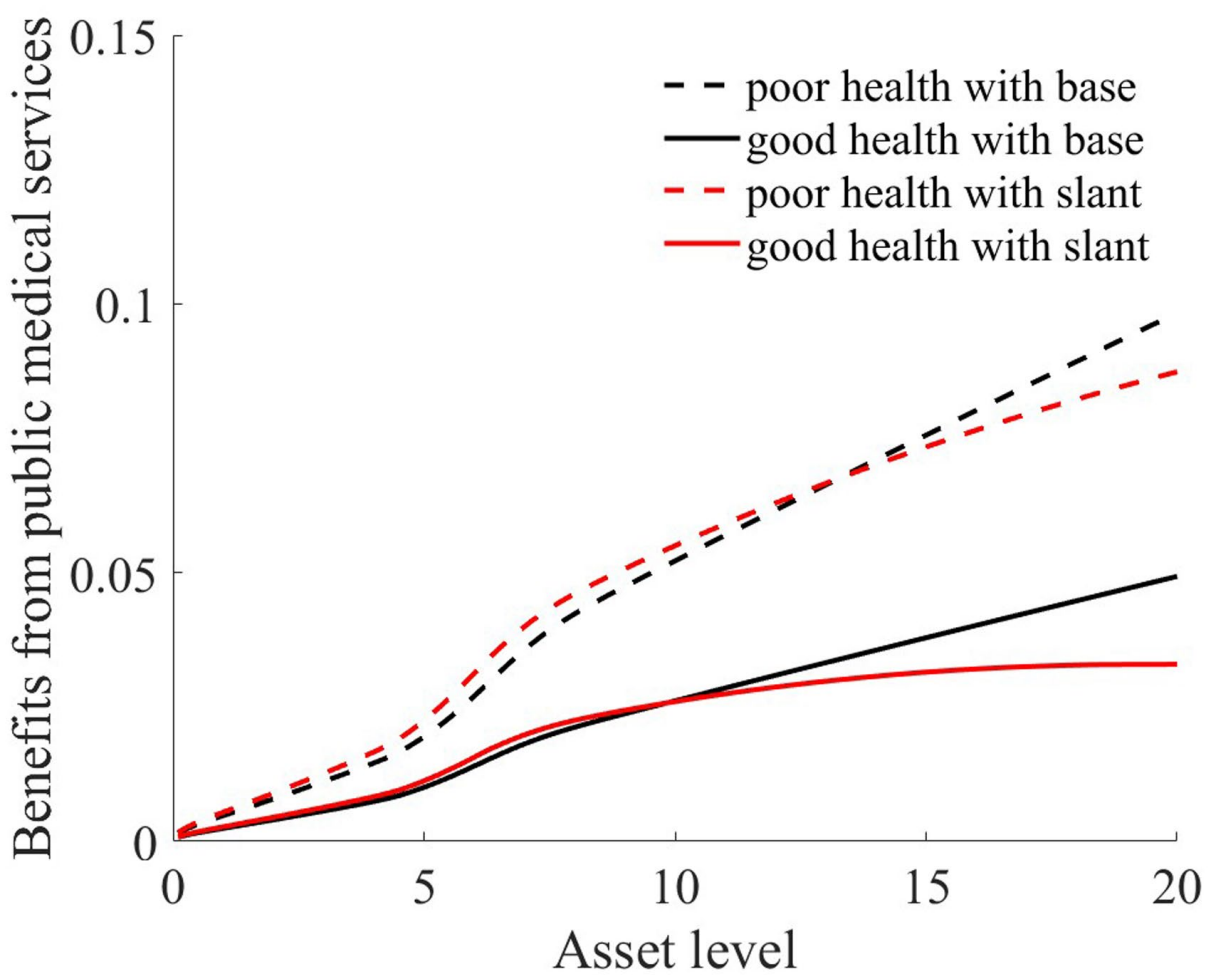
Benefits from public medical services.

**Figure 9:**
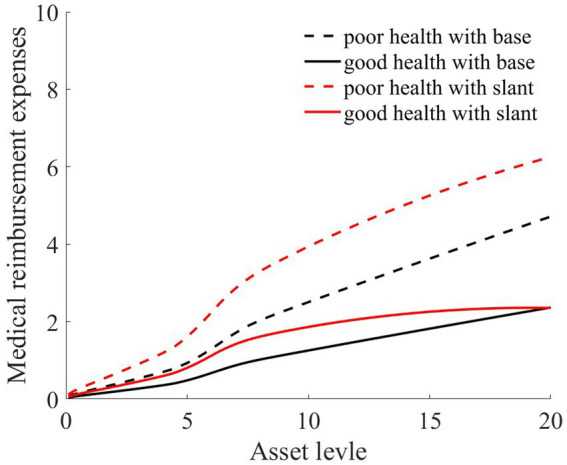
Medical reimbursement expenses.

Consistent with the previous section, we simulated the scenarios of 100,000 households. We also presented the benefit statuses of rural households in two health conditions: good health and poor health. Based on Eqs 6, 7, we calculated the benefit values of rural households with different asset levels and health statuses. Additionally, we computed the reimbursement expenses for medical insurance, which are depicted in Figures 8, 9.

As Figures 8, 9 illustrate, the solid black line represents rural households with good health conditions, while the dashed black line signifies those with poorer health conditions. Both curves demonstrate a consistent trend: the higher the asset level of the household is, the more it benefits from medical services (Figure 8). This is because, despite every household having the same medical insurance premium obligations, those with higher asset levels tend to have higher medical expenditures. Under a proportional compensation policy, they receive higher reimbursements from their medical insurance (Figure 9), thereby accruing greater benefits from medical services. This essentially results in a “reverse redistribution” from households with lower assets to those with higher assets. Simultaneously, a larger proportion of government fiscal subsidies flow to households with higher asset levels. This phenomenon of reverse wealth distribution intensifies wealth inequality among rural households.

Medical insurance provides insured households with a proportionate reimbursement, elevating their asset levels after a health shock. This, in turn, raises their shadow price of assets (as shown in Figure 7), especially for households with poorer health conditions. This incentivizes these households to make greater agricultural production investments, facilitating rapid capital accumulation toward a higher stable equilibrium and preventing these households from falling into poverty traps (as depicted in Figure 6). Thus, both the investment incentive and compensation effects of medical insurance serve to reduce wealth inequality (as measured by the Gini coefficient and Theil index).

**Figure 10:**
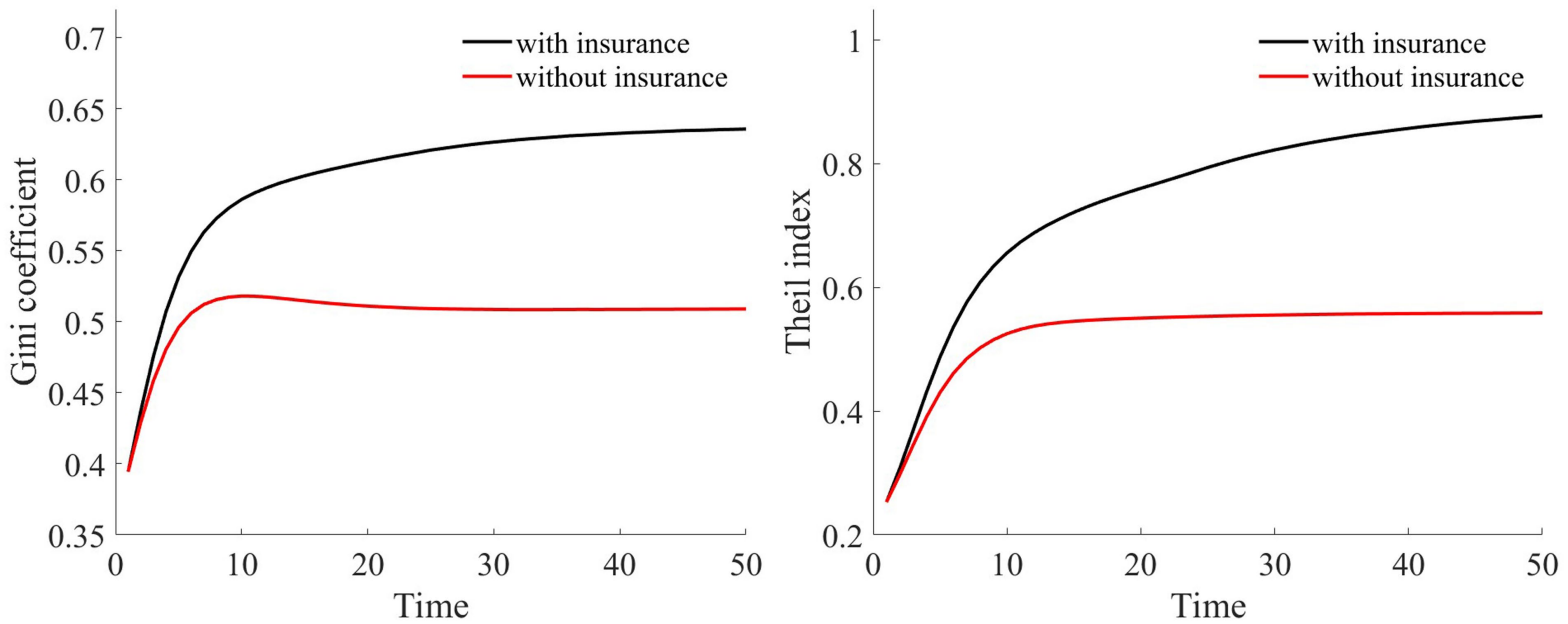
Changes in wealth inequality indices.

In summary, due to uniform medical insurance premiums and proportionate medical expense reimbursements, medical insurance tends to favor households with higher wealth over those with lower wealth, thereby exacerbating the wealth disparities between households. However, the investment incentive effect of medical insurance boosts agricultural production investments among rural households, enabling them to accumulate capital more swiftly and thereby mitigating wealth inequality. Overall, medical insurance contributes to a reduction in wealth inequality (as illustrated in Figure 10).

### 3.4 Analysis of the effects of dual-slanted reimbursement policies

#### 3.4.1 Impact of the slanted compensation policy on poverty vulnerability

If the medical insurance compensation ratio were to be universally increased for all households (potentially even to 100%), this might render the fiscal policy untenable. Moreover, since households with higher wealth exhibit more substantial spending power, a uniform increase in the reimbursement rate for all could intensify the “reverse redistribution” effect.

**Figure 11:**
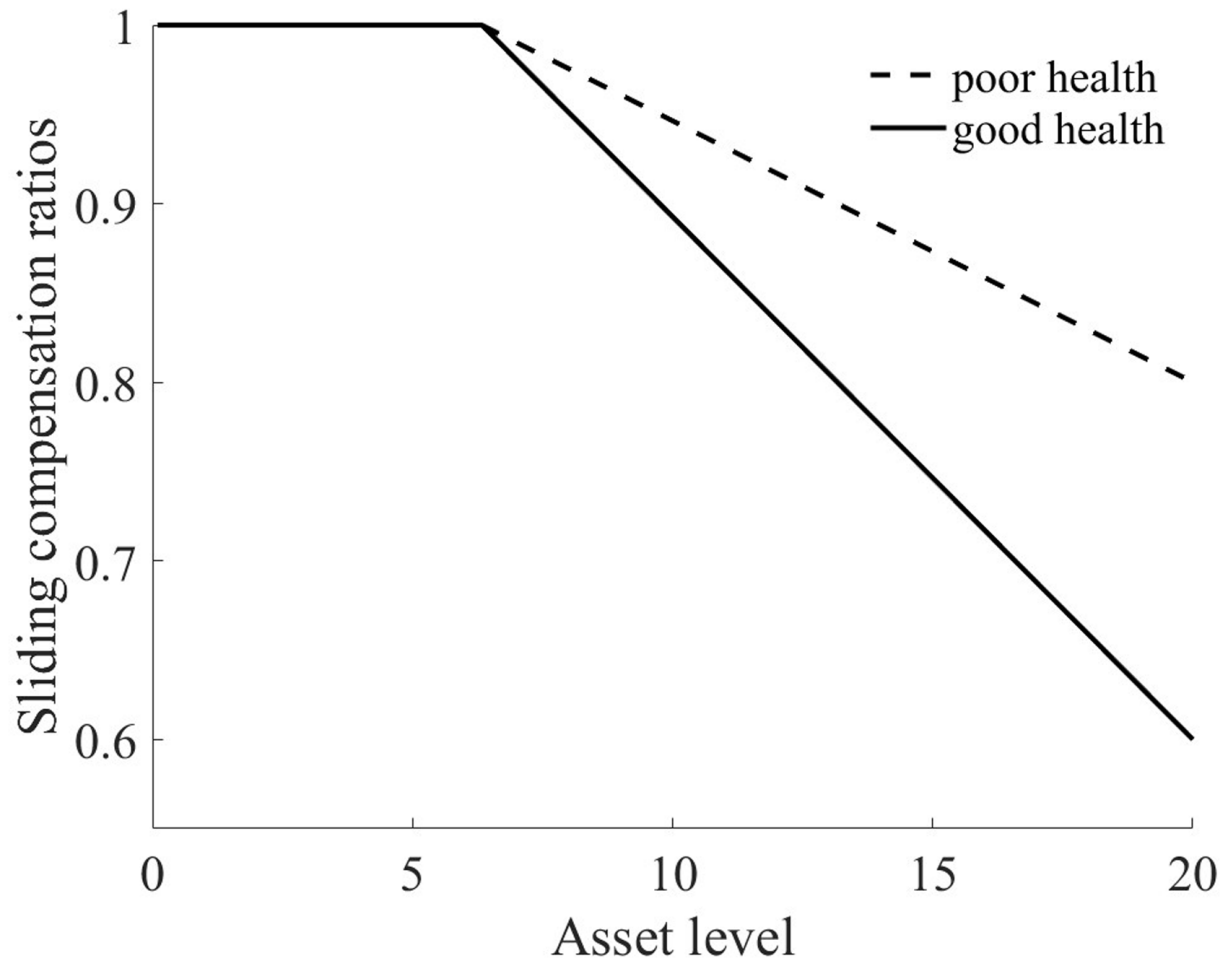
Changes in sliding compensation ratios.

Therefore, with a nuanced approach, we tailored a sliding compensation policy targeting both the wealth and health dimensions to alleviate this reverse redistribution challenge stemming from medical insurance. As illustrated in Figure 11, for rural households situated below the Micawber threshold, we peg the compensation ratio at 100%. However, for those surpassing the Micawber threshold, the medical cost compensation ratio diminishes linearly as wealth levels increase. This structured approach particularly aids rural households in poorer health by preferentially increasing their compensation ratios, thereby mitigating their medical expense burdens.

Upon the introduction of the sliding medical insurance compensation policy, there is a noticeable uptick in the reimbursement amounts for households within the middle to lower wealth. Specifically, for individuals in poorer health, the elevated medical insurance compensation ratios (as depicted in Figure 11) lead to increased medical expense reimbursements. This further alleviates the medical financial burdens borne by such rural households.

**Figure 12:**
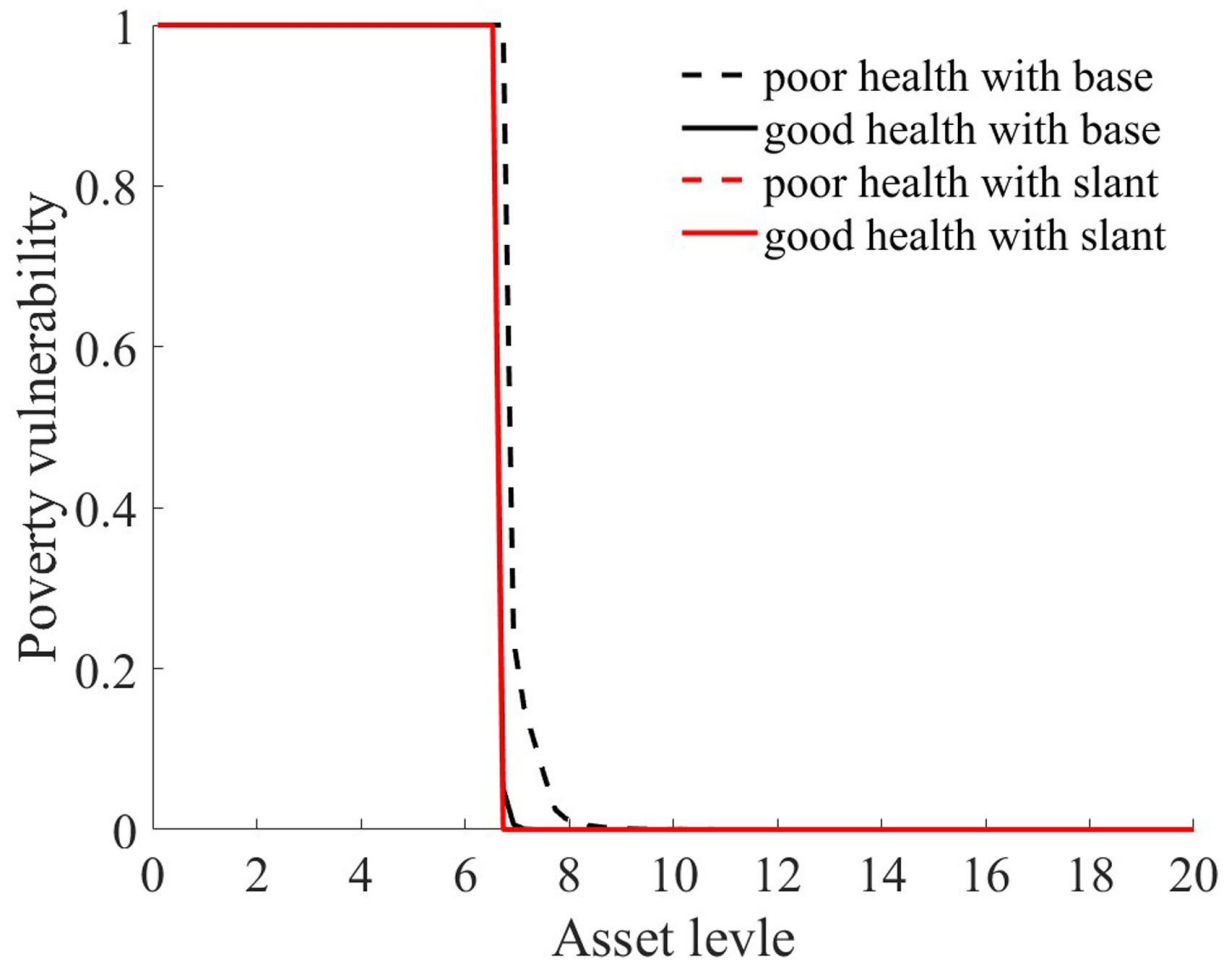
Poverty vulnerability.

In the wake of health risk, the wealth disparity between these households and households with better health conditions is reduced. This results in a marked diminution of wealth inequalities exacerbated by health issues. Ultimately, the poverty vulnerability of residents with poor health is mitigated. As shown in Figure 12,^7^ the poverty vulnerability of both types of households converges, becoming more aligned.

#### 3.4.2 The impact of the slanted compensation policy on wealth inequality

As shown in Figures 8, 9, the red line represents the situation under the slanted compensation policy, while the black line denotes the baseline scenario (proportional compensation policy). The solid and dashed lines distinguish between individuals in good health and those in poor health, respectively. In comparison to the proportional medical insurance compensation policy, the slanted compensation approach enhances the benefit levels for middle-and low-wealth households. This is because these households, under a more generous medical insurance compensation scheme, receive increased compensation. However, for these middle-to low-wealth households, even with an increased medical insurance compensation rate, their limited capacity for medical expenditure places them at a disadvantage in receiving medical services when compared to high-wealth households (as indicated in Figures 8, 9).

Relative to those in good health, households with poorer health and with middle to low wealth benefit the most from the slanted compensation policy. This slanted compensation strategy adjusts the benefit distribution for households with better financial standing, redirecting more public resources toward middle-and low-wealth households. Especially for households with poor health, the double-tilted compensation approach ensures that rural households with low wealth utilize more public medical service resources. This strategy further reduces the “reverse redistribution” phenomenon where middle-to low-wealth households subsidize their wealthier counterparts.

**Figure 13:**
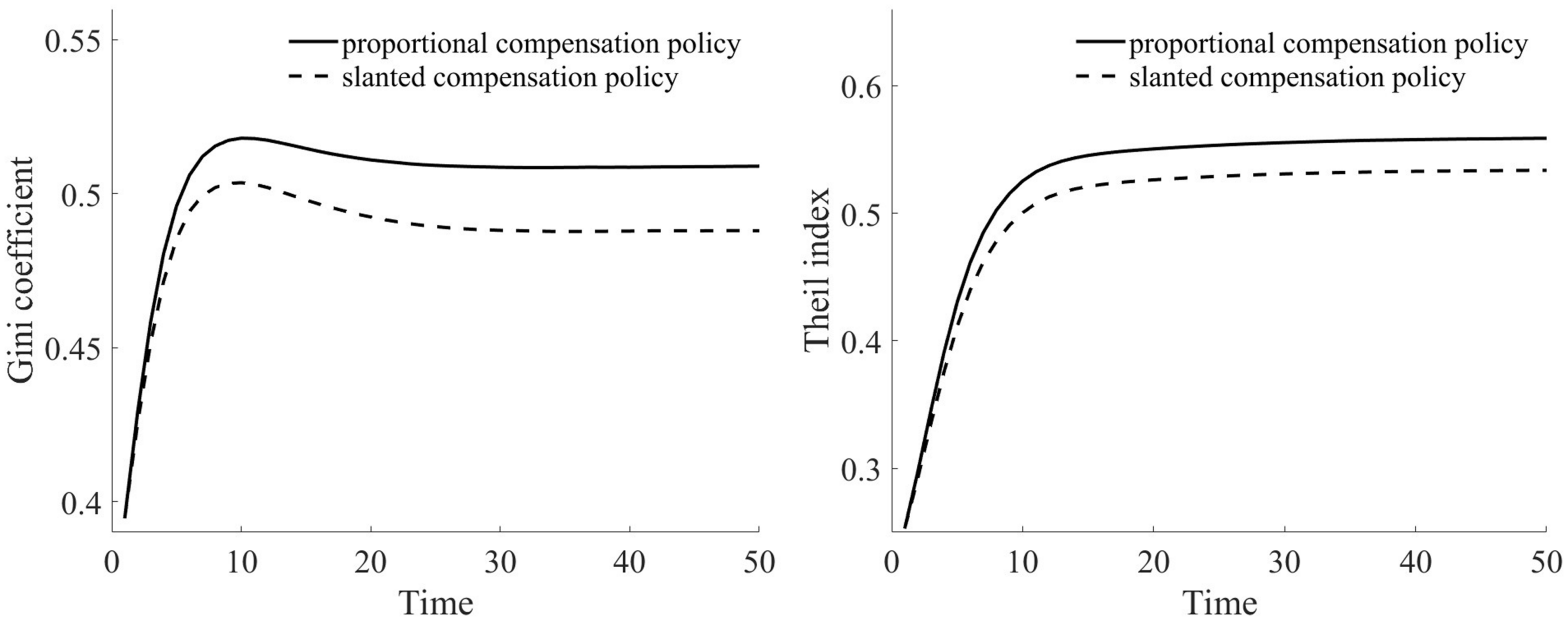
Wealth inequality indices under different compensation structures.

Owing to the increased reimbursement rates for both middle-to-low wealth households and those with poorer health conditions, there is a clear reduction in vulnerability to poverty for these groups. This reduces their likelihood of falling into poverty traps, as depicted in Figure 12. Such a scenario implies that these households can invest more substantially in agricultural production, consistently accumulate assets, and eventually gravitate toward a high-stable equilibrium. As a consequence, there is also a subsequent reduction in both the Theil index and the Gini coefficient, diminishing the level of wealth inequality among rural households, as illustrated in Figure 13.

## 4 Conclusions and policy recommendations

This study integrates heterogeneous health risks and medical insurance into a multi-equilibrium framework. By employing the value function iteration algorithm, we computed the policy functions for consumption and assets. Through stochastic simulations, we examined the impact of heterogeneous health risks, medical insurance, and a dual-slanted compensation policy on vulnerability to poverty and wealth inequality among rural households.

Our findings reveal that under the influence of heterogeneous health risks, vulnerability to poverty and wealth inequality among rural households are significantly heightened. Notably, households with poorer health conditions exhibit considerably greater vulnerability to poverty than do their counterparts with better health conditions. With the introduction of medical insurance, due to the investment incentive effect and the compensation mechanism of medical insurance, there is a marked reduction in the vulnerability of households to poverty (with a more pronounced effect for those with poorer health conditions) as well as in the wealth inequality between households. Owing to uniform premium payments and proportional medical expense reimbursements, a “reverse redistribution” phenomenon occurs from low-wealth households to high-wealth households, exacerbating wealth inequality. Overall, medical insurance mitigates both the vulnerability to poverty and wealth inequality among rural households.

In light of the “reverse redistribution” effect caused by medical insurance, this study introduces a dual-slanted medical insurance compensation policy designed around both wealth and health. We discuss the impact of this policy on vulnerability to poverty and wealth inequality. For rural households with poorer health conditions, the slanted compensation approach significantly alleviates their medical financial burdens, reducing their vulnerability to poverty. Concurrently, this approach addresses the wealth “reverse redistribution” issue arising from medical insurance, enhancing the efficiency of public medical service utilization. Ultimately, this results in a decrease in wealth inequality.

This study provides several insights for public health insurance practitioners. (1) The government should invest in establishing a comprehensive and efficient health record system to collect and analyze the health data of rural household members. This approach will provide crucial support for the formulation and implementation of health insurance policies, ensuring that the policies are more precise and effective than those currently in use. (2) Differential medical insurance policies should be designed based on the wealth and health status of households. Households with poor health and lower wealth should be provided with higher medical expense compensation and lower insurance costs. (3) Dual-slanted compensation policies should be implemented within the existing medical insurance system, offering varying levels of compensation based on household wealth and health status. This approach will help alleviate the financial burden of low-income households while reducing the wealth inequality caused by insurance mechanisms. (4) Investment in public medical services in rural areas should be increased, and basic medical infrastructure should be improved. This can enhance access to medical services for low-income households in rural areas, reducing poverty risks due to health hazards. (5) The government should regularly monitor and evaluate the effectiveness of medical insurance policies, especially their impact on low-income and health-challenged households, and adjust and optimize based on evaluation results.

Furthermore, the government should continuously explore and innovate with the implementation methods for dual-slanted compensation policies to adapt to socioeconomic changes and advancements in medical technology in the future. This includes but is not limited to optimizing compensation mechanisms, expanding insurance coverage, enhancing public health investment, and improving policy transparency and public participation.

The implementation of these suggestions is expected to reduce the poverty risks associated with health hazards, decrease wealth inequality, and enhance the overall welfare of rural families. These measures will also lay the groundwork for the long-term development and innovation of dual-slanted compensation policies. The research in this paper helps alleviate the issue of high medical costs for rural families and provides effective suggestions for consolidating achievements in poverty alleviation, thus achieving rural revitalization and shared prosperity.

## Data availability statement

The raw data supporting the conclusions of this article will be made available by the authors, without undue reservation.

## Author contributions

XZ: Conceptualization, Funding acquisition, Investigation, Writing – original draft. XY: Conceptualization, Formal analysis, Methodology, Software, Writing – review & editing.

## Footnotes

^1^According to the current standards set by the World Bank, China has eradicated absolute poverty. For more details, please refer to: https://www.worldbank.org/content/dam/Worldbank/document/WB-goals2013.pdf and https://www.un.org/en/global-issues/ending-poverty.

^2^The data is sourced from: https://www.gov.cn/xinwen/2021-11/16/content_5651269.htm.

^3^Previous models on multiple equilibria have primarily discussed the impact of asset risks and agricultural risks on the vulnerability to poverty of rural households (25–27). This paper builds upon these foundations with certain expansions. Furthermore, the existence of poverty traps has been thoroughly discussed in studies by Carter and Barrett (28), Thomas and Gaspart (29), Toth (30), and Radosavljevic et al. (31). This topic is not reiterated in our paper.

^4^Notably, such a nonconvex production function does not necessarily lead to poverty traps (32, 33). This paper investigates only scenarios that include poverty traps.

^5^In 2016, the New Cooperative Medical Scheme was integrated with the Urban Resident Medical Insurance to form the Urban and Rural Residents’ Basic Medical Insurance. Given that the current insurance coverage rate is close to 100%, this paper does not discuss the impact of insurance demand.

^6^Please refer to the “National Medical Security Development Statistical Bulletin” published by China’s National Medical Security Administration, http://www.nhsa.gov.cn/.

^7^In this paper, the red dashed line in Figure 12 represents the poverty vulnerability of households with poor health status under the dual-slanted compensation policy. Notably, under this policy, households with both good and poor health have the same level of poverty vulnerability. Therefore, the red dashed line and the red solid line overlap.
